# Disruptive variants of *CSDE1* associate with autism and interfere with neuronal development and synaptic transmission

**DOI:** 10.1126/sciadv.aax2166

**Published:** 2019-09-25

**Authors:** Hui Guo, Ying Li, Lu Shen, Tianyun Wang, Xiangbin Jia, Lijuan Liu, Tao Xu, Mengzhu Ou, Kendra Hoekzema, Huidan Wu, Madelyn A. Gillentine, Cenying Liu, Hailun Ni, Pengwei Peng, Rongjuan Zhao, Yu Zhang, Chanika Phornphutkul, Alexander P. A. Stegmann, Carlos E. Prada, Robert J. Hopkin, Joseph T. Shieh, Kirsty McWalter, Kristin G. Monaghan, Peter M. van Hasselt, Koen van Gassen, Ting Bai, Min Long, Lin Han, Yingting Quan, Meilin Chen, Yaowen Zhang, Kuokuo Li, Qiumeng Zhang, Jieqiong Tan, Tengfei Zhu, Yaning Liu, Nan Pang, Jing Peng, Daryl A. Scott, Seema R. Lalani, Mahshid Azamian, Grazia M. S. Mancini, Darius J. Adams, Malin Kvarnung, Anna Lindstrand, Ann Nordgren, Jonathan Pevsner, Ikeoluwa A. Osei-Owusu, Corrado Romano, Giuseppe Calabrese, Ornella Galesi, Jozef Gecz, Eric Haan, Judith Ranells, Melissa Racobaldo, Magnus Nordenskjold, Suneeta Madan-Khetarpal, Jessica Sebastian, Susie Ball, Xiaobing Zou, Jingping Zhao, Zhengmao Hu, Fan Xia, Pengfei Liu, Jill A. Rosenfeld, Bert B. A. de Vries, Raphael A. Bernier, Zhi-Qing David Xu, Honghui Li, Wei Xie, Robert B. Hufnagel, Evan E. Eichler, Kun Xia

**Affiliations:** 1 Center for Medical Genetics and Hunan Key Laboratory of Medical Genetics, School of Life Sciences, Central South University, Changsha, Hunan, China.; 2 Department of Genome Sciences, University of Washington School of Medicine, Seattle, WA, USA.; 3 Institute of Life Sciences, Key Laboratory of Developmental Genes and Human Disease, Southeast University, Nanjing, Jiangsu, China.; 4 Department of Neurobiology, Beijing Key Laboratory of Neural Regeneration and Repair, Beijing Laboratory of Brain Disorders (Ministry of Science and Technology), Beijing Institute of Brain Disorders, Capital Medical University, Beijing, China.; 5 Key Laboratory of Developmental Disorders in Children, Liuzhou Maternity and Child Healthcare Hospital, Liuzhou, Guangxi, China.; 6 Division of Human Genetics, Warren Alpert Medical School of Brown University, Hasbro Children's Hospital/Rhode Island Hospital, Providence, RI, USA.; 7 Department of Clinical Genetics, Maastricht University Medical Center, Maastricht, Netherlands.; 8 Department of Pediatrics, University of Cincinnati College of Medicine, Division of Human Genetics, Cincinnati Children’s Hospital, Cincinnati, OH, USA.; 9 Institute for Human Genetics and Department of Pediatrics, University of California, San Francisco, San Francisco, CA, USA.; 10 GeneDx, Gaithersburg, MD, USA.; 11 University Medical Center Utrecht, Utrecht, Netherlands.; 12 Department of Pediatrics, Xiangya Hospital, Central South University, Changsha, China.; 13 Department of Molecular and Human Genetics, Baylor College of Medicine, Houston, TX, USA.; 14 Department of Molecular Physiology and Biophysics, Baylor College of Medicine, Houston, TX, USA.; 15 Department of Clinical Genetics, Erasmus MC University Medical Center, Rotterdam, Netherlands.; 16 Goryeb Children’s Hospital, Atlantic Health System, Morristown, NJ, USA.; 17 Department of Molecular Medicine and Surgery, Center for Molecular Medicine, Karolinska Institute, Stockholm, Sweden.; 18 Department of Clinical Genetics, Karolinska University Hospital, Stockholm, Sweden.; 19 Department of Neurology, Kennedy Krieger Institute, Baltimore, MD, USA.; 20 Program in Human Genetics, Johns Hopkins School of Medicine, Baltimore, MD, USA.; 21 Oasi Research Institute–IRCCS, Troina, Italy.; 22 School of Medicine and the Robinson Research Institute, University of Adelaide at the Women’s and Children’s Hospital, Adelaide, South Australia, Australia.; 23 Adult Genetics Unit, Royal Adelaide Hospital, and School of Medicine, University of Adelaide, Adelaide, South Australia, Australia.; 24 Department of Pediatrics, University of South Florida, Tampa, FL, USA.; 25 Division of Medical Genetics, Children’s Hospital of Pittsburgh of UPMC, Pittsburgh, PA, USA.; 26 Central Washington Genetics Program, Virginia Mason Memorial, Yakima, WA, USA.; 27 Children Development Behavior Center of the Third Affiliated Hospital of Sun Yat-sen University, Guangzhou, Guangdong, China.; 28 Mental Health Institute of the Second Xiangya Hospital, Central South University, Changsha, Hunan, China.; 29 Baylor Genetics, Houston, TX, USA.; 30 Department of Human Genetics, Donders Institute for Brain, Cognition and Behaviour, Radboud University Medical Center, Nijmegen, Netherlands.; 31 Department of Psychiatry, University of Washington, Seattle, WA, USA.; 32 Ophthalmic Genetics and Visual Function Branch, National Eye Institute, NIH, Bethesda, MD, USA.; 33 Howard Hughes Medical Institute, University of Washington, Seattle, WA, USA.; 34 Key Laboratory of Medical Information Research, Central South University, Changsha, Hunan, China.; 35 CAS Center for Excellence in Brain Science and Intelligences Technology (CEBSIT), Chinese Academy of Sciences, Shanghai 200030, China.; 36 Hunan Key Laboratory of Animal Models for Human Diseases, Central South University, Changsha, Hunan 410078, China.

## Abstract

RNA binding proteins are key players in posttranscriptional regulation and have been implicated in neurodevelopmental and neuropsychiatric disorders. Here, we report a significant burden of heterozygous, likely gene-disrupting variants in *CSDE1* (encoding a highly constrained RNA binding protein) among patients with autism and related neurodevelopmental disabilities. Analysis of 17 patients identifies common phenotypes including autism, intellectual disability, language and motor delay, seizures, macrocephaly, and variable ocular abnormalities. HITS-CLIP revealed that Csde1-binding targets are enriched in autism-associated gene sets, especially FMRP targets, and in neuronal development and synaptic plasticity–related pathways. Csde1 knockdown in primary mouse cortical neurons leads to an overgrowth of the neurites and abnormal dendritic spine morphology/synapse formation and impaired synaptic transmission, whereas mutant and knockdown experiments in *Drosophila* result in defects in synapse growth and synaptic transmission. Our study defines a new autism-related syndrome and highlights the functional role of CSDE1 in synapse development and synaptic transmission.

## Teaser

CSDE1 disruptive mutations are associated with autism.

## INTRODUCTION

Autism spectrum disorder (ASD) is a group of neurodevelopmental disorders (NDDs) with considerable genetic and clinical heterogeneity (*1*). The core behavioral abnormalities are characterized by impairments in social communication and interaction, restricted interests, and repetitive behaviors, as defined in the Diagnostic and Statistical Manual of Mental Disorders, Fifth Edition (DSM-5) (*2*). In addition to the core symptoms, language delay and intellectual disability (ID) are two of the most common co-occurring conditions. Genetic studies have implicated hundreds of susceptibility genes (https://gene.sfari.org/) (*3*), and de novo variants in more than 100 genes confer increased risk for ASD (*4*–*6*). Even so, these genes account for only a small fraction of patients (*7*), suggesting that a large number of genes with extremely rare variants await discovery as study sample sizes increase (*5*).

Enrichment and pathway analyses have shown that ASD risk genes are frequently associated with transcriptional regulation, especially targets of RNA binding proteins (RBPs) (*5*, *6*, *8*). RBPs mainly function in posttranscriptional gene regulation, which is essential to sustain cellular metabolism, coordinating maturation, transport, stability, and degradation of all classes of RNAs (*9*). In particular, two RBPs have been linked to ASD and NDDs, namely, RBFOX1 (*10*) and FMRP (*11*) encoded by *FMR1*, the fragile X syndrome (FXS) gene. Identification of these genes and their RNA binding targets has been important in the identification of ASD disease networks and potential therapeutic interventions (*10*, *11*).

*CSDE1*, also known as *UNR*, encodes a highly conserved RBP containing five cold-shock domains and has been implicated in both neuronal migration and differentiation (*12*, *13*). Our previous genome-wide association study suggested *CSDE1* as a potential ASD risk gene (*14*). Using a targeted sequencing approach and with collaboration of multiple international centers, we found and report on the phenotypes of 18 patients with likely gene-disrupting (LGD) variants in *CSDE1*, identifying a new ASD-related syndrome. High-throughput sequencing of RNA isolated by cross-linking immunoprecipitation (HITS-CLIP) analyses show that Csde1-binding targets are significantly enriched in ASD-related genes and significantly overlap FMRP and RBFOX targets. In vitro and in vivo functional analyses highlight the important role of Csde1 in neuronal development and synaptic transmission.

## RESULTS

### *CSDE1* disruptive variants associate with ASD and related NDDs

**Fig. 1:**
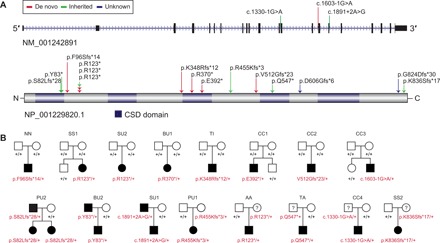
Spectrum of *CSDE1* LGD variants and patient facial features. (**A**) Diagram of the canonical *CSDE1* isoform (NM_001242891.1 and NP_001229820.1). The locations of LGD variants are indicated. (**B**) Pedigrees of eight families with de novo LGD variants (above) and eight families with transmitted LGD variants (below). Carrier parents or sibling in at least four families (PU2, BU2, SU1, and PU1) are affected or show substantial family history.

We initially targeted the coding region of *CSDE1* using a modified single-molecule molecular inversion probe (smMIP) approach (Materials and Methods) among 4045 ASD probands from the Autism Clinical and Genetic Resources in China (ACGC) cohort and identified three de novo LGD variants (two nonsense and one canonical splice site) from two simplex quad families and one trio family (CC1.p1, CC2.p1, CC3.p1; Table 1 and Fig. 1). We applied the chimpanzee-human divergence model (CH model) (*15*) to calculate the excess probability of de novo LGD variants within *CSDE1* (Materials and Methods) and observed that the probability of detecting three or more de novo LGD variants within *CSDE1* in the ACGC cohort is significant (*P* = 1.98 × 10^−7^, binomial test) even after genome-wide multiple testing correction (*P*_adj_ = 0.004, binomial test, Bonferroni correction).

**Table 1:** Summary of *CSDE1* LGD variants. Isoform, NM_001242891. BCM, Baylor College of Medicine; WES, whole-exome sequencing; gDNA, genomic DNA; cDNA, complementary DNA; SSC, Simons Simplex Collection.

**Patient ID**	**Cohort**	**Method**	**Function**	**Cohort size**	**Variant in gDNA (hg19,chr1)**	**Variant in cDNA**	**Protein change**	**Inheritance**
PU2.p1	Providence	WES	Stopgain	–	g.115282407_115282407delG	c.243_243delC	p.S82Lfs*28	Paternal
BU2.p1	Bethesda	WES	Stopgain	–	g.115282401_115282402insT	c.248_249insA	p.Y83*	Paternal
NN1.p1	Nijmegen	WES	Frameshift	2,418	g.115282363delA	c.287_287delT	p.F96Sfs*14	De novo
SU2.p1	San Francisco	WES	Stopgain	–	g.115280664G>A	c.367C>T	p.R123*	De novo
SS1.p1	SSC	WES	Stopgain	2,508	g.115280664G>A	c.367C>T	p.R123*	De novo
AA.p1	Adelaide	Target	Stopgain	10,745	g.115280664G>A	c.367C>T	p.R123*	Maternal
TI.p1	Troina	Target	Frameshift	10,745	g.115275369_115275370TT	c.1043_1044delAA	p.K348Rfs*12	De novo
BU1.p1	Baltimore	WGS	Stopgain	29	g.115275305G>A	c.1108C>T	p.R370*	De novo
CC1.p1	ACGC	Target	Stopgain	4,045	g.115275239C>A	c.1174G>T	p.E392*	De novo
CC4.p1	Changsha	Target	Splicing	10,745	g.115273044C>T	c.1330-1G>A	–	Paternal
PU1.p1	BCM	WES	Frameshift	8,910	g.115273009_115273010insT	c.1363_1364insA	p.R455Kfs*3	Maternal
CC2.p1	ACGC	Target	Frameshift	4,045	g.115269672_115269673insC	c.1533_1534insG	V512Gfs*23	De novo
CC3.p1	ACGC	Target	Splicing	4,045	g.115269008C>T	c.1603-1G>A	–	De novo
TA.p1	TASC	Target	Stopgain	10,745	g.115268971G>A	c.1639C>T	p.Q547*	Paternal
NN2.p1	Nijmegen	WES	Frameshift	2,418	g.115267916_115267917insC	c.1816_1817insG	p.D606Gfs*6	Not maternal
SU1.p1	BCM	WES	Splicing	8,910	g.115267840T>C	c.1891+2A>G	–	Paternal
TU.p1	BCM	WES	Frameshift	8,910	g.115261250_115261250delC	c.2471_2471delG	p.G824Dfs*30	Not maternal
SS2.p1	Swedish	Target	Frameshift	10,745	g.115260816_115260819delTCTT	c.2506_2509delAAGA	p.K836Sfs*17	Maternal

On the basis of this observation, we targeted *CSDE1* for sequencing in a larger international cohort of patients in the Autism Spectrum/ID network (Materials and Methods). We identified and validated five additional LGD variants (AA.p1, CC4.p1, SS2.p1, TA.p1, and TI.p1; Table 1 and Fig. 1). In this cohort, four of the five variants were inherited and only one was a de novo variant. Unfortunately, no detailed clinical follow-up or neuropsychiatric assessment could be performed on the carrier parents in this subset. Last, by leveraging the web-based platform GeneMatcher (*16*) and personal communications with our collaborators, we collected 10 additional patients (NN1.p1, NN2.p1, PU1.p1, PU2.p1, BU1.p1, BU2.p1, SS1.p1, SU1.p1, SU2.p1, and TU.p1; Table 1 and Fig. 1) with *CSDE1* LGD variants and neurodevelopmental phenotypes (table S1). Four are de novo, three are inherited, and, in two, the father’s DNA was not available. Each of the carrier parents and sibling either exhibited mild neurodevelopmental phenotypes or presented with substantial family history of ASD or developmental disability (DD) (Fig. 1B). The carrier father in family PU2, for example, was previously diagnosed with global developmental delay, and the sibling who also carries this variant has a history of ASD, seizures, and anxiety. The carrier father in family BU2 presented with a history of language and motor delays, suspected ID, and macrocephaly—features also observed in the proband. The carrier father in family SU1 has a specific learning disability. Family PU1 has substantial maternal family history of epilepsy and anxiety disorder (extended family members not tested) (Fig. 1B). In addition to LGD variants, we also collected three patients with de novo missense variants (table S1) through this effort.

In total, we identified 18 families with *CSDE1* LGD variants, including eight de novo, eight inherited, and two with undetermined inheritance (Table 1 and Fig. 1). We observed one CpG-mediated recurrent site of variant (p.R123*) identified in three independent families: two de novo and one inherited. On the basis of all patient data, we estimated genome-wide significance of the genetic findings. First, we identified seven patients (NN1.p1, SS1.p1, TI.p1, BU1.p1, CC1.p1, CC2.p1, and CC3.p1; Table 1) with de novo LGD variants from cohorts, for a total of 19,745 individuals. These data suggest a significant excess of de novo LGD variants after genome-wide multiple testing correction (*P*_adj_ = 3.6 × 10^−9^, binomial test, Bonferroni correction) (Materials and Methods). Second, *CSDE1* is highly intolerant to variants, as predicted by the probability of loss-of-function intolerance score (pLI = 1.00) (*17*) and the residual variation intolerance score (%RVIS = 6.18) (*18*). No LGD variants are present in the 1000 Genomes Project or the Exome Sequencing Project; only one LGD variant was identified in 45,375 Exome Aggregation Consortium (ExAC) non-neuropsychiatric samples (*19*). We estimated the mutational burden of 15 *CSDE1* LGD variants screened from cohorts with a total of 28,655 probands versus 45,375 ExAC non-neuropsychiatric subset samples (Materials and Methods). By this metric, probands show a significant burden of LGD variants regardless of inheritance status [odds ratio (OR), 22.2; *P* = 1.6× 10^−5^, two-tailed Fisher’s exact test]. We note that the mean sequence coverage of *CSDE1* exons is more than 40-fold (fig. S1), so this difference is unlikely the result of low sequence coverage in ExAC. These data strongly support the association of both inherited and de novo LGD variants in *CSDE1* with ASD and related NDDs.

### Disruption of *CSDE1* defines a new ASD-related syndrome in early development

Through patient recontact and a review of the available clinical records, we assembled a phenotypic description for 17 probands ranging in age from 3 to 19 years old (average age of 8.5 years) (Table 2 and table S1). Only one of the probands was an adult. The most consistent phenotypes include ASD, ID, delayed speech, and delayed motor development (Table 2). Of the 15 individuals with ASD evaluation, 10 were formally diagnosed with ASD, whereas 1 had clinical impression of ASD. Although ASD assessment was not attempted in the other two patients, autistic features including repetitive behavior were reported for one of them (Table 2). Of the 16 individuals for whom cognitive ability was assessed, 14 had a diagnosis of mild-to-severe ID and the remaining 2 patients had cognitive performance in the below-average range of intellectual functioning. All 17 probands had delay in speech, and motor delay was observed in 15 of 17 individuals for whom information was available.

**Table 2:** Genotype-phenotype correlations of 17 probands with *CSDE1* LGD variants. +, present; −, absent; blank, not reported. DN, de novo; MI, maternal inheritance; PI, paternal inheritance; NMI, not maternal inheritance; EEG, electroencephalographic.

**Patient ID**	**PU2.p1**	**BU2.p1**	**NN1.p1**	**SS1.p1**	**SU2.p1**	**AA.p1**	**TI.p1**	**BU1.p1**	**CC1.p1**	**CC4.p1**	**PU1.p1**	**CC2.p1**	**CC3.p1**	**TA.p1**	**SU1.p1**	**TU.p1**	**SS2.p1**	**Total**
Variant inheritance	PI	PI	DN	DN	DN	MI	DN	DN	DN	PI	MI	DN	DN	PI	PI	NMI	MI	8 DN,8 INH
Age at last examination (years)	4.5	3.5	12	17	7	19	11	13	8	5	8.4	3	3.8	7.9	5.2	5.9	10	3–19
Sex	F	M	M	F	F	M	M	F	M	M	F	M	M	M	M	M	F	11 M, 6 F
Neurodevelopmental problems																		
Developmental delay (speech)	+	+	+	+	+	+	+	+	+	+	+	+	+	+	+	+	+	17/17
Developmental delay (motor)	+	+	−	+	+	+	+	+	+	+	+	+	+	+	+	+	−	15/17
ASD/autistic features*	−	+	+	+	−	−	±	+	+			+	+	+	+	−	+	11/15
ID^†^	+	+	+	+	±	+	+	+	+	+	+	±	+	+	+	+	±	14/16
Neurological problems																		
Epilepsy/seizure^‡^	−	−	−	−	−	±	+	±	±	+	+	±	−	−		−	−	7/16
EEG abnormalities	+		−	+	−	+	+	−	−	+	+	−	−					6/12
MRI brain abnormalities	+	+	−	−	+	−	+	+	−	+		−	−			−	+	7/14
Macrocephaly	+	+	−	+	−	+	−	−	−	−		+	−		+	−	−	6/14
Sleep disturbances	−	−	+	+	−	−	−	−	+	−		−	−			−	+	4/14
Behavior problems																		
Repetitive behavior	+	+	+	+	−	+	+	+	+	+		+	+	+	+	−	+	14/15
ADHD	+		−	+	+	−	−	+	+		+	+	+			−	+	9/13
Anxiety	+	+	−	+	+	−	+	−	+			+	−			−	−	7/13
Obsessive behavior	−	+	−	−	−	+	+	−	+			−	−			−	−	4/13
Self-injurious behavior	−	−	−		−	−	+	+	−	+		−	−				−	3/12
Aggressive behavior	−	−	−	−	−	−	+	+	−	−		−	−			−	−	2/14
Systemic problems																		
Eye abnormalities^§^		+	+		+	−	+	+	+	−	+	−	−			−	−	7/13
Recurrent infections	−	−	−	+	−	−	−	+	+			+	+			+	−	6/13
Hypotonia	+	+	−	+	−	−	+	+	−		+	−	−				−	6/13
Hand deformity^║^	+	+	+	+	−	−	−	−	−	−	+	−	−			+	−	6/15
Short stature	+	+	−	−	+	−	−	−	−	−	−	−	−		−	−	−	3/16

*+, ASD; ±, autistic features.

†+, mild to severe ID; ±, below average or learning disability.

‡+, epilepsy; ±, seizure but no epilepsy diagnosis.

§Eye abnormalities are variable (see table S1).

║Hand deformity including brachydactyly (4), polydactyly (1), and clinodactyly (1).

In addition, we noted several common comorbidities in probands with *CSDE1* LGD variants (Table 2). Seven of 16 patients (44%) had recurrent seizures or epilepsy. Six of 14 patients (43%) reported increased head circumference or macrocephaly. Seven of 14 patients (50%) presented with abnormalities on brain magnetic resonance imaging (MRI). Anxiety behavior (7 of 13, 54%) and parent-reported attention-deficit/hyperactivity disorder (ADHD) (9 of 13, 69%) were also highly associated. In addition to neurological and behavior problems, several common systemic problems were also observed. Variable ocular abnormalities were present in 7 of 13 patients (54%), including iris coloboma, hyperopia, and strabismus. Six of 15 patients (40%) had hand findings, including 4 with brachydactyly, 1 with fifth finger clinodactyly, and 1 with polydactyly. Six of 13 patients (46%) had hypotonia.

The only adult proband (AA.p1) identified in this study showed apparently substantial progress in the management of neurodevelopmental issues since childhood and now presented with good social skills and milder ID. Instead, the individual has substantial psychiatric difficulties described as mild depression as a teenager and a formal diagnosis of schizophrenia at the age of 18 years.

### Csde1 RNA binding targets enriched in ASD-related gene sets

To identify the RNA targets of Csde1 in the nervous system, we performed HITS-CLIP using mouse whole brain (Materials and Methods). We performed two independent experiments that showed a median correlation of *R* = 0.43 (fig. S2A). We applied two software programs, ABLife and Piranha, to call the binding peaks in each experiment (Materials and Methods). We found that Csde1 binds mRNA across the gene body, including the coding sequence, 3′ untranslated region, and intronic regions. Of the known targets of mammalian Csde1, we identified *Csde1* and *Pabpc1* mRNAs in both independent experiments. We subsequently selected 26 NDD-associated genes identified in both experiments or by both calling programs for validation by RNA immunoprecipitation sequencing (RIP-seq). We validated 76.9% of these targets (table S2). Although invalidated genes do not necessarily imply false positive binding, we report a higher validation rate (14 of 16, 87.5%) in genes identified by both peak-calling programs from both experiments (table S2). Applying this criterion yields 210 targets, of which 184 have human homologs (table S3).

**Fig. 2:**
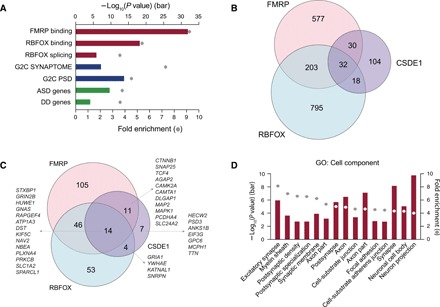
Enrichment analyses of Csde1 RNA binding targets. (**A**) Bar plot shows Csde1-binding targets significantly enriched in seven ASD-related gene sets, especially the FMRP RNA binding targets. (**B**) Venn diagram shows the overlap of the RNA binding targets of CSDE1 and other ASD-associated RBPs (FMRP and RBFOX). (**C**) Venn diagram shows the overlap of the SFARI genes that are RNA binding targets of CSDE1, FMRP, and RBFOX, with gene names indicated. (**D**) Bar plot shows Csde1-binding targets enriched in neuronal development– and synapse development–related cell components [Fisher’s exact test, false discovery rate (FDR)–corrected]. Top 15 significant cell components are shown.

Using this high-confidence gene set, we tested whether Csde1-binding targets are enriched in ASD-associated genes. We considered seven ASD-associated gene sets: (i) FMRP RNA binding targets (*11*), (ii) RBFOX (RBFOX1/2/3) RNA binding targets (*10*), (iii) RBFOX splicing targets (*10*), (iv) ASD-associated genes [Simons Foundation Autism Research Initiative (SFARI)], (v) DD-associated genes (*20*), (vi) genes encoding postsynaptic density proteins (*21*), and (vii) genes involved in the synaptome (*21*). We revealed that Csde1-binding targets are significantly enriched in all gene sets (Fig. 2A). We observed a greater than threefold enrichment with ASD-associated (OR, 3.79; *P* = 5.47 × 10^−10^, two-tailed Fisher’s exact test) and DD-associated (OR, 3.66; *P* = 2.38 × 10^−4^, two-tailed Fisher’s exact test) genes (Fig. 2A). Unexpectedly, Csde1-binding targets most strongly overlap the targets of other ASD-related RBPs, FMRP (OR, 9.22; *P* = 2.18 × 10^−32^, two-tailed Fisher’s exact test) and RBFOX (OR, 5.36; *P* = 5.45 × 10^−18^, two-tailed Fisher’s exact test) (Fig. 2A). Among 184 Csde1 targets, 80 (43.5%) are overlapped with either FMRP targets (*n* = 62) or RBFOX targets (*n* = 50) (Fig. 2B). Notably, among the 36 SFARI genes that Csde1 binds, 29 (80.5%) are overlapped with either FMRP (*n* = 25) or RBFOX (*n* = 18) targets (Fig. 2C). Gene Ontology and Kyoto Encyclopedia of Genes and Genomes (KEGG) pathway analyses revealed that Csde1-binding targets associate with synapse development and plasticity and with neuronal development–related cell components and pathways (Fig. 2D and fig. S2B).

### Disruption of Csde1 interferes with synapse development and synaptic transmission in mouse cortical pyramidal neurons

CSDE1 is widely expressed in different human tissues (*22*). To better understand the spatiotemporal expression pattern of CSDE1, we further investigated CSDE1 mRNA expression pattern using RNA sequencing data from the Human Brain Transcriptome database (http://hbatlas.org/) (*23*). CSDE1 mRNA is expressed in a variety of different areas of the brain, showing the highest level of expression during embryogenesis, decreasing slightly postnatally but maintaining a high level across the whole lifespan (fig. S3A). We also assessed protein expression patterns by Western blot using embryonic and early postnatal mouse brains. Similar to the human mRNA expression pattern, the Csde1 protein is highly expressed in the cortex and cerebellum during the embryonic and postnatal stages of mouse development (fig. S3B).

**Fig. 3:**
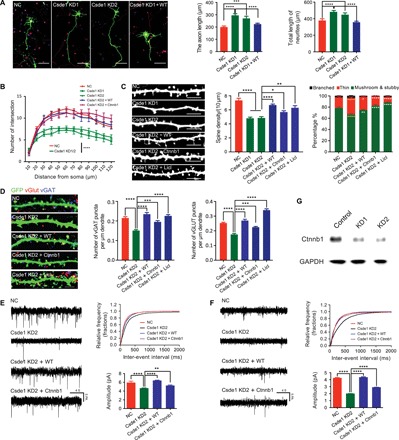
Disruption of Csde1 interferes with neuronal development. (**A**) Csde1 KD promoted neurite and axon growth (NC, 73 neurons; Csde1 KD1, 60 neurons; Csde1 KD2, 53 neurons; Csde1 KD1 + WT, 82 neurons). Neurons were colabeled with 4′,6-diamidino-2-phenylindole (DAPI) (nuclei), green fluorescent protein (GFP) (overall neuronal morphology), and SMI 312 (axon). Scale bar, 100 μm. (**B**) Csde1 KD reduced the complexity of dendritic arborization (NC, 32 neurons; Csde1 KD1, 21 neurons; Csde1 KD2, 42 neurons; Csde1 KD2 + WT, 27 neurons; Csde1 KD2 + Ctnnb1, 26 neurons). (**C**) Csde1 KD disrupted dendritic spine morphogenesis and maturation (NC, 27 neurons; Csde1 KD1, 41 neurons; Csde1 KD2, 23 neurons; Csde1 KD2 + WT, 40 neurons; Csde1 KD2 + Ctnnb1, 28 neurons; Csde1 KD2 + Licl, 23 neurons). Scale bar, 10 μm. (**D**) Csde1 KD reduced the number of excitatory (vGlut) and inhibitory (vGAT) synapses (NC, 41 neurons; Csde1 KD2, 46 neurons; Csde1 KD2 + WT, 30 neurons; Csde1 KD2 + Ctnnb1, 39 neurons; Csde1 KD2 + Licl, 28 neurons). Scale bar, 10 μm. (**E** and **F**) Voltage-clamp whole-cell recordings showed that both frequency and amplitude of mEPSCs and mIPSCs were decreased in Csde1 KD neurons (three neurons for each condition). (**G**) Immunoblot showed that β-catenin expression was markedly decreased in Csde1 KD neurons. Statistical data were presented as means ± SEM. *P < 0.05, **P < 0.01, ***P < 0.001, ****P < 0.0001.

We next investigated whether Csde1 is required for neuronal and synapse development. Because Csde1 is highly expressed in the cortex and most of the disease-associated *CSDE1* variants are predicted or validated (fig. S4) to result in loss of function, we designed two distinct short hairpin RNAs (shRNAs) to knock down (KD) Csde1 in mouse primary cultures of cortical neurons. Endogenous Csde1 expression was markedly reduced after transfection of neurons with these two shRNAs (fig. S5). Transferring the shRNAs at day 0 in vitro (DIV0) and analyzing neurites at day 5 (DIV5), we observed a marked increase in both the total neurite length and axonal length in Csde1 KD neurons (Fig. 3A). In addition, we observed reduced dendritic complexity in the Csde1 KD neurons at DIV14 (Fig. 3B). To examine the role of Csde1 on dendritic spine development and morphogenesis, we examined the secondary dendrites of Csde1 KD neurons at DIV18 and observed that Csde1 KD neurons generated fewer dendritic spines compared with controls (Fig. 3C), consistent with fewer synaptic contacts. These differences were accompanied by thin or branched spines, consistent with an immature spine morphology (Fig. 3C). These results suggest that Csde1 is essential for normal development of axons and dendritic spines.

We also performed immunostaining using antibodies to synaptic markers vGlut (excitatory) and vGAT (inhibitory). We found that the numbers of both excitatory and inhibitory synapses were markedly reduced in Csde1 KD neurons compared with controls (Fig. 3D). Coexpression of human CSDE1 (hCSDE1) rescued axonal, dendritic, and synaptic phenotypes (Fig. 3, A to D). Last, we performed whole-cell electrophysiology from neurons transfected with either control or shRNA to examine whether Csde1 KD affects excitatory and inhibitory neurotransmission. Both the frequency and amplitude of miniature excitatory postsynaptic currents (mEPSCs) and miniature inhibitory postsynaptic currents (mIPSCs) were significantly decreased in Csde1 KD neurons (Fig. 3, E and F) when compared to controls. Again, coexpression of hCSDE1 rescued the abnormal neurotransmission phenotypes (Fig. 3, E and F).

Our HITS-CLIP and RIP-seq experiments identified *Ctnnb1* mRNA as a binding target of Csde1 (table S2). β-Catenin, encoded by *Ctnnb1*, is the key downstream component of the canonical Wnt signaling pathway and is critical for neuronal dendritic morphogenesis (*24*). To investigate the potential molecular mechanisms underlying the deficient dendritic spine phenotypes observed in Csde1 KD neurons, we assayed β-catenin mRNA and protein expression level in Csde1 KD neurons. Immunoblot experiments showed that β-catenin protein expression was markedly reduced in Csde1 KD neurons (Fig. 3G), although mRNA expression levels were not significantly altered. These data are consistent with previous reports that Csde1 regulates expression at the posttranscriptional level (*25*). We then expressed a degradation-resistant β-catenin mutant construct alongside Csde1 KD in mouse cortical neurons. Expression of stabilized β-catenin rescued the abnormal spine density and morphology and neurotransmission defects associated with Csde1 disruption (Fig. 3, B to F). To determine whether dendritic spine and synapse phenotypes arise from decreased Wnt/β-catenin signaling efficiency in Csde1 KD neurons, we sought to rescue neurodevelopmental phenotypes by stimulating the pathway upstream of the putative pathway block. As anticipated, treatment with the upstream ligand lithium rescued the abnormal spine density and morphology and neurotransmission (Fig. 3, C and D).

Together, these results indicate that Csde1 is essential for proper neuronal and synapse development. Specifically, disruption of Csde1 causes neurite overgrowth, abnormal dendritic spine morphology, impaired synaptogenesis, and impaired neurotransmission in mouse primary cultures of cortical neurons. Csde1 disruption suppresses *Ctnnb1* expression, which, in turn, contributes to abnormal dendritic spine morphogenesis and synapse development.

### Disruption of Csde1 interferes with synapse growth and neurotransmission in vivo

**Fig. 4:**
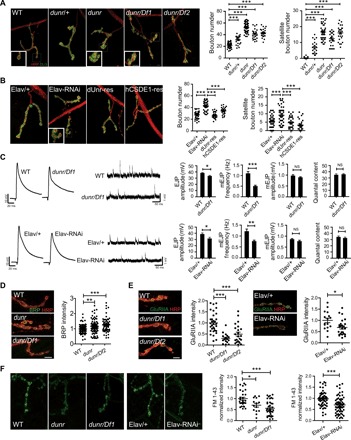
Disruption of dUnr interferes with synapse development and transmission in *Drosophila*. (**A**) Left: Representative NMJ4 synapses of dUnr-related lines (WT, *dunr*/+, *dunr*, *dunr/Df1*, and *dunr/Df2*). Right: Both bouton number and satellite bouton number were increased in *dunr/+* line (*n* = 22), *dunr* line (*n* = 58), *dunr/Df1* line (*n* = 23), and *dunr/Df2* line (*n* = 25) compared to control (*n* = 61). The boutons were costained with anti-HRP labeling the neuronal plasma membrane (red) and anti-DLG (green) labeling a postsynaptic scaffold protein. Magnified image of the boxed region at left bottom shows the terminal bouton or the satellite bouton. Scale bar, 5 μm. (**B**) Left: Representative NMJ4 morphology of the Elav/+ line, a pan-neuronal dUnr KD line (Elav-RNAi), a *Drosophila* UNR rescue line (dUnr-res), and a human CSDE1 rescue line (hCSDE1-res). Right: Both bouton number and satellite bouton number were increased in Elav-RNAi line (*n* = 45) compared to Elav/+ line (*n* = 45). In dUnr-res line (*n* = 34) and hCSDE1-res line (*n* = 28), the numbers were decreased compared to Elav-RNAi line (*n* = 45). Scale bar, 5 μm. (**C**) Left: Representative traces of EJPs and mEJPs in the indicated genotypes. Right: The amplitudes of EJPs were mildly decreased in both *dunr/Df1* line (WT: *n* = 9; *dunr/Df1*: *n* = 13) and pan-neuron KD line (Elav/+: *n* = 29; Elav-RNAi: *n* = 17). Slightly but not significantly decreased mEJP amplitude and no significant change of quantal content were observed on both KD and *dunr/Df1* lines. NS, not significant. (**D**) The normalized fluorescent intensity of BRP was slightly increased in *dunr* (*n* = 76) and *dunr/Df1* (*n* = 63) lines compared to controls. Scale bar, 5 μm. (**E**) The normalized fluorescent intensity of postsynaptic GluRIIA was markedly reduced in *dunr/Df1* (*n* = 18), *dunr/Df2* (*n* = 23), and KD lines (*n* = 25) compared to controls. Scale bar, 5 μm. (**F**) Decreased normalized fluorescent intensity of FM 1-43 dye was detected in *dunr* (*n* = 14), *dunr/Df1* (*n* = 40), and KD (*n* = 61) lines compared to controls. Scale bar, 5 μm. Statistical data were presented as means ± SEM. *P < 0.05, **P < 0.01, and ***P < 0.001.

The *Drosophila* neuromuscular junction (NMJ) provides a valuable tool for studying genes associated with NDDs. A homolog of human CSDE1 (hCSDE1), named *Drosophila* Upstream of N-ras (dUnr), is highly conserved at the protein level, sharing most functional domains with hCSDE1 (*22*, *25*). We collected and tested several fly strains relating to dUnr, including a hypomorphic mutant (*dunr*) and two deficiency strains that removed dUnr (*Df1* and *Df2*) (fig. S6). NMJ morphological analysis at muscle 4 was performed on the wild-type (WT) and dUnr strains by dissecting third-instar wandering larvae and labeling NMJs with anti–horseradish peroxidase (HRP) antibody and anti-discs large (DLG) antibodies, which mark the neuronal membranes and the postsynaptic membranes of type Ib boutons, respectively. We observed a significant increase of both total bouton number and satellite bouton number in dUnr homozygote mutants (*dunr*) (Fig. 4A), with heterozygous mutants (*dunr/+*) showing a more modest but significant increase. Two lines of dUnr hemizygous mutants, *dunr/Df1* and *dunr/Df2*, demonstrated identical synaptic overgrowth phenotypes, thereby minimizing effects of genetic background (Fig. 4A).

To clarify whether disruption of dUnr in the nervous system contributes to the defects of NMJs, we established a dUnr KD line using UAS-dUnr-RNAi constructs (fig. S6) driven by pan-neuronal Elav-Gal4. Neuronal KD of dUnr mimicked dUnr mutant phenotypes, resulting in a significant increase in both total and satellite bouton numbers (Fig. 4B). To eliminate the possibility of off-target effects from the KD line, we coexpressed an RNA interference (RNAi)–resistant dUnr (dUnr-res), which rescued the phenotypes (Fig. 4B). Synaptic overgrowth induced by neuronal KD can be suppressed by coexpression of hCSDE1 (hCSDE1-res) (Fig. 4B), indicating that the function of hCSDE1 and dUnr is conserved during synapse development. Considering the intriguing overlap of binding targets between Csde1 and FMRP and that overexpression of dFmrp in *Drosophila* has inverse or gain-of-function phenotypes as compared with loss of function, we tested whether overexpression of dUnr or hCSDE1 also presents inverse phenotypes when compared with loss of dUnr. Unfortunately, there is no significant difference for both bouton number and satellite bouton number in dUnr or hCSDE1 overexpression lines driven by pan-neuronal Elav-Gal4 (fig. S7).

To assess the functional consequences of disrupted synapse development, we performed electrophysiological analyses of NMJs at muscle 6/7 of the third-instar larvae using intracellular recordings at 0.6 mM Ca^2+^. We recorded the evoked excitatory junction potentials (EJPs), which reflect the evoked transmitter release, and the miniature EJPs (mEJPs), which reflect the spontaneous transmitter release. We observed a mild decrease of the EJP amplitudes and mild but not significant decrease of mEJP amplitudes in both *dunr/Df1* and Elav-RNAi lines compared with the controls (Fig. 4C). There was no significant alteration of quantal contents in both lines, indicating no or very minor effect of dUnr on synaptic efficacy (Fig. 4C). Although the mEJP amplitude was not significantly altered, the mEJP frequency was markedly decreased in both lines (Fig. 4C), indicating impaired synaptic transmission in NMJs. We then performed immunostaining for the presynaptic active zone component Bruchpilot (BRP) and the postsynaptic glutamate receptor IIA (GluRIIA). We observed a slight increase of BRP fluorescent intensity (Fig. 4D) and a marked decrease of GluRIIA fluorescent intensity (Fig. 4E). Reduced mEJP frequency is often associated with the reduced BRP. However, we observed the opposite. Changes in mEJP amplitude are often attributable to changes in glutamate receptor. Although we observed a marked decrease of GluRIIA intensity, the mEJP amplitude only showed very mild decrease. One underlying explanation is that the compensatory mechanisms in *Drosophila* larval NMJs may adjust presynaptic and postsynaptic responses to maintain synaptic transmission efficacy, which have been observed in previous studies (*26*–*28*). These results suggest that loss of dUnr disrupts normal synapse development and function.

Considering that abnormal satellite boutons usually reflect defective synaptic endocytosis, we asked whether disruption of CSDE1 causes abnormal synapse endocytosis. We labeled the synaptic vesicle with FM 1-43 dye, which can be taken up in a synaptic vesicle by endocytosis, in *dunr/Df1* and the KD lines. We observed a significant reduced amount of loaded FM 1-43 dye in both *dunr/Df1* and the KD lines (Fig. 4F), indicating a potential role of dUnr in synaptic endocytosis.

## DISCUSSION

We report a significant association of both de novo and inherited truncating variants in *CSDE1* among pediatric patients with autism and more broadly defined NDDs. In total, we present detailed clinical and genetic information for 17 patients and observe several consistent phenotypes, including ASD, mild-to-severe ID, and speech and motor developmental delay. In addition, seizures, macrocephaly, anxiety, ADHD, hypotonia, and ocular defects were also associated with *CSDE1* LGD variants. The common phenotypes observed in the probands suggest a new ASD-related syndrome defined by *CSDE1* LGD variants.

Although LGD variants are nearly absent from population controls, it is interesting that in half the cohort, *CSDE1* truncating variants are inherited from a parent. Although learning disabilities, delayed development, and reduced cognitive ability were noted in some of the carrier parents, transmitting parents generally presented with less severe phenotypes than the affected children. Similarly, our single adult patient was higher functioning but did experience psychiatric issues later in life, including a diagnosis of schizophrenia. The basis for this phenotypic variability is unknown and may reflect modifying genetic factors or, alternatively, the condition may change over time, improving in certain facets and worsening with others at different neurodevelopmental stages throughout life. Longitudinal studies of *CSDE1* families, including more detailed phenotypic and genetic assessment of carriers, are needed to better understand the progression and risk factors associated with variable expression of this disorder across individuals and age.

It is well known that variants in two RBP-encoded genes, *FMR1* and *RBFOX1*, cause ASD and other NDDs (*29*, *30*). Our study presents a new highly constrained RBP gene, *CSDE1*, causing a new ASD-related syndrome. RBP targets are important predictors of disease pathology, and the targets of both FMRP and RBFOX have already been well established (*10*, *11*). In this study, we focused on identifying the targets of Csde1 binding in the mouse brain and found a large overlap with those of FMRP and RBFOX. Similarly, we find that Csde1-binding targets are significantly enriched in ASD-associated genes, including dozens of high-risk ASD genes, such as *GRIN2B*, *STXBP1*, *GRIA1*, *CTNNB1*, *SNAP25*, and *TCF4*, as well as synapse development and plasticity-related cell components or pathways. It should be noted that several well-defined ASD genes, which did not make our high-confidence set based on overlap between independent experiments, were also validated as Csde1-binding targets, including *SYNGAP1*, *DYRK1A*, *TNRC6B*, and *NCKAP1*. Therefore, it is critical to design additional more stringent studies to further delineate the full spectrum of Csde1 targets.

RBPs regulate almost all steps of RNA expression, including transcription, alternative splicing, and translation (*31*). Although there is an intriguing overlap of CSDE1 and FMRP targets, based on the current knowledge, the underlying regulation mechanisms of CSDE1 and FMRP appear to differ. First, CSDE1 regulates mRNA stability and/or translation by interacting with distinct complexes mainly in the cytoplasm (*22*). A paradigmatic case is the regulation of internal ribosome entry site–mediated translation (*32*). In comparison, FMRP is associated with suppressed messenger ribonucleoprotein particles and actively translating polyribosomes during translation (*33*, *34*) and is believed to specifically bind to mRNAs and regulate their translation. Second, in many cases, loss of FMRP-mediated regulation leads to increased translation and protein levels of the targets. Our study shows that loss of Csde1 leads to a suppression of Ctnnb1 protein levels. Third, from the functional perspective, dendritic spine density is increased on cortical neurons in both FXS humans and Fmr1 knockout mice (*35*, *36*). Excitatory junction current amplitudes were significantly elevated in dFmrp null mutants (*37*). However, in this study, we observed decreased spine density in mouse cortical neurons and mildly decreased EJP amplitudes in KD and mutant *Drosophila*. Moreover, overexpression of FMRP leads to inverse or gain-of-function phenotypes as compared to the loss of FMRP in many instances. In this study, we did not observe synapse overgrowth in the CSDE1 overexpression line when compared to KD or mutant lines, where both bouton number and satellite bouton number decreased. One possibility may be an antagonistic functional relationship between CSDE1 and FMRP, although further functional studies will be required to test this hypothesis.

Several studies have recently reported that disruption of CSDE1 leads to defects in normal neural development at a variety of levels. Kobayashi and colleagues, for example, found Csde1 expressed in migrating precerebellar neurons, and shRNA-mediated inhibition resulted in a failure of precerebellar neurons to complete migration to their prospective target regions. This suggested a crucial role for Csde1 in the proper control of both radial and tangential migration of precerebellar neurons (*12*). Another recent study reported that CSDE1 is highly expressed in human embryonic stem cells (hESCs), where it serves to maintain their undifferentiated state, preventing a default neural differentiated state. It was suggested that CSDE1 serves as a central posttranscriptional regulator of hESC identity and neurogenesis (*13*).

In the current study, we observed abnormal spine development in Csde1 KD neurons with an overall decrease in spine density and the prevalence of filopodia-like immature spines. Structural changes of dendritic spines are correlated with functional changes of neural circuitry and synapse plasticity. We found that Csde1 deficiency consistently suppresses excitatory and inhibitory synaptic transmission in cultured neurons. In the *Drosophila* model, we found that disruption of dUnr led to impaired NMJ synapse growth, as indicated by increased total synaptic and satellite bouton number. These morphological abnormalities were accompanied by defect synapse endocytosis and impairment in synaptic function. Presynaptic KD of dUnr mimicked these phenotypes, and synaptic overgrowth could be suppressed by coexpression of dUnr and hCSDE1. Our results argue that dUnr and hCSDE1 play an important role in synapse development and function. Specifically, we propose that synaptic maturation is dependent on CSDE1 activity, and loss-of-function variants of *CSDE1* contribute to clinical outcomes via defects in synaptic connectivity and transmission.

Our data further suggest that Csde1 positively regulates β-catenin in mouse cortical neurons at the posttranscriptional level. The binding of CSDE1 with β-catenin was also reported in a recent study that investigated the CSDE1 targets in melanoma cell (*38*). Depletion of CSDE1 in melanoma cells reduced CSDE1 expression at the protein level, which is consistent with our results. Moreover, the reduced CSDE1 expression resists the presence of proteasome inhibitors, suggesting that CSDE1 may regulate β-catenin expression at the translational level (*38*). In this study, expression of β-catenin or activation of the Wnt signaling pathway rescues these Csde1 deficiency defects. In addition to β-catenin, we also found that Csde1 binds another important Wnt signaling pathway–related gene, *TCF4*, which functions as one of the major transcriptional mediators of the canonical Wnt signaling pathway. Because β-catenin is critical for dendritic morphogenesis (*24*) and defects in Wnt/β-catenin signaling have been strongly implicated in the development of ASD (*39*), we hypothesize that Csde1 regulates dendritic spine maturation and synapse function, in part, through regulation of the Wnt/β-catenin signaling pathway.

In summary, through international collaboration and targeted sequencing of large patient cohorts, we identified an excess of LGD variants in a highly conserved RBP-encoded gene, *CSDE1*, and defined a new ASD-related syndrome. Although little is known to date about the function of CSDE1 in neurodevelopment, our HITS-CLIP and in vitro and in vivo functional analyses indicate that CSDE1 plays an essential role in neuronal and synapse development, including synapse transmission. The significant overlap of RNA binding targets between CSDE1 and FMRP suggests that behavioral and potential therapeutic interventions (*40*) for FXS are also likely to benefit patients with CSDE1-related syndrome.

## MATERIALS AND METHODS

### Study cohorts and targeted sequencing

We initially targeted 4045 ASD probands from the Chinese population [ACGC cohort described previously (*41*)] for *CSDE1* sequencing. ACGC patients were diagnosed primarily according to DSM-4 and/or DSM-5 criteria, documenting additional comorbid conditions where possible. Peripheral blood DNA from all patients with ASD and their parents, where available, was collected after obtaining informed consent. Genomic DNA was extracted from whole blood using a standard proteinase K digestion and phenol-chloroform method. In the second stage, targeted sequencing was performed on a larger international cohort [described elsewhere (*42*)] with 10,745 patients with a primary diagnosis of ASD and/or ID/DD. Informed consents from all participants were obtained. This study was approved by the Institutional Review Board (IRB) of Central South University.

Targeted sequencing of *CSDE1* was performed using smMIP technology—a highly cost-effective targeted sequencing method (*15*). In brief, MIPs were designed using MIPgen with an updated scoring algorithm. Amplification of the captured DNA was performed as previously reported (*41*). Libraries were sequenced using the Illumina HiSeq 2000 platform. Clean reads were aligned against hg19 (GRCh37 reference genome) with BWA-MEM (v0.7.13) (*43*) after removing incorrect read pairs and low-quality reads. Single-nucleotide variants and indels were called with FreeBayes (v0.9.14) (*44*). Variants exceeding 10-fold sequence coverage and read quality more than 20 (QUAL > 20) were annotated with SeattleSeq (*45*) Annotation 138 using reference GRCh37/hg19. LGD variants and rare missense variants in *CSDE1* (minor allele frequency < 1% in ExAC) were selected for validation using Sanger sequencing in both patients and parents where available.

### Statistical analyses for *CSDE1* LGD variants

De novo significance was calculated using a binomial model that incorporates gene-specific variant rates estimated from an overall rate of variation in coding sequences and estimates the relative locus-specific rates based on the CH model (*15*) with an expected rate of 1.5 de novo variants per exome. *P* values were corrected genome-wide for the number of genes (18,946). Burden of *CSDE1* LGD variants between patients and ExAC nonpsychiatric samples was performed using Fisher’s exact test. All statistical analyses were performed using the R statistical language (v3.2.4) (www.r-project.org/).

### HITS-CLIP experiments using mouse brain

Two independent Csde1 HITS-CLIP experiments were performed with the mixture of three whole-brain tissue lysates of 15-day-old (P15) mice using the standard protocols. Tissues were triple washed with ice-cold phosphate-buffered saline (PBS), and ultraviolet (UV) cross-linking was performed with UV irradiation type C (254 nm) at 400 mJ/cm^2^. Samples were ground, and cell lysis was performed in cold lysis buffer (1× PBS, 0.1% SDS, 0.5% NP-40, and 0.5% sodium deoxycholate) supplemented with a 1% ribonuclease inhibitor (Takara, Kusatsu, Shiga, Japan) and 2% protease inhibitor cocktail (Roche, Basel, Switzerland) for 30 min. Cell lysates were cleared by centrifugation at 10,000 rpm for 10 min at 4°C, and the supernatants were used for immunoprecipitation. Study protocols comply with all relevant ethical regulations and were approved by the IRB of Central South University. All animal experiments were approved by the Institutional Animal Care and Use Committee of the Central South University.

For DNA digestion, RQ1 (Promega, Madison, WI; final concentration, 0.05 U/μl) was added to the lysate and incubated at 37°C for 3 min. RNA digestion was performed by adding micrococcal nuclease (1:10,000, Thermo Fisher Scientific, Waltham, MA), followed by incubation at 37°C for 10 min. For immunoprecipitation, 600 μl of lysate was incubated with 15 μg of antibody or control immunoglobulin G (IgG) antibody overnight at 4°C. The immunoprecipitates were further incubated with protein A/G Dynabeads for 2 to 3 hours at 4°C. After applying to a magnet and removing the supernatants, the beads were sequentially washed with wash buffer (1× PBS, 1% SDS, 0.5% NP-40, and 5% sodium deoxycholate), high-salt wash buffer (5× PBS, 1% SDS, 0.5% NP-40, and 5% sodium deoxycholate), and PNK (polynucleotide kinase) buffer [50 mM tris (pH 7.4), 10 mM MgCl_2_, and 0.5% NP-40] twice.

After washing with PNK buffer as described above, dephosphorylation and phosphorylation were performed with FastAP thermosensitive alkaline phosphatase (Thermo Fisher Scientific, Waltham, MA) and T4 polynucleotide kinase (Thermo Fisher Scientific, Waltham, MA). The immunoprecipitated protein-RNA complex was eluted from the beads by heat denaturing and resolved on a Novex 4-12% Bis-Tris precast polyacrylamide gel (Invitrogen, Carlsbad, CA). The protein-RNA complexes were cut from the gel (Invitrogen, Carlsbad, CA), and RNA was extracted with TRIzol after digesting the proteins. The recovered RNA was used to generate libraries with a TruSeq small RNA library preparation kit (Gnomegen, San Diego, CA) following the manufacturer’s instructions. Libraries corresponding to 150 to 250 base pairs (bp) were purified and quantified. The libraries were sequenced on the Illumina NextSeq 500 system with 151-bp paired-end reads by ABlife Inc. (Wuhan, China).

### HITS-CLIP data analyses

Raw reads were first discarded if containing more than 2-N bases, then reads were processed by clipping adaptor and removing low-quality bases, and too short reads (less than 13 nucleotides) were also dropped. FASTX-Toolkit (version 0.0.13) was used to obtain a clean set of reads. Clean reads were mapped to the GRCm38.p3 version of the mouse genome by TopHat2 (*46*). After removing duplicate reads, the remainder was used for subsequent analyses.

### CLIP sequencing peak calling

Two software programs, Piranha and Ablife, were used to perform peak calling. Piranha has been described elsewhere (*47*). The Ablife analyses are summarized as follows. After alignment, identical aligned reads were counted and merged as unique reads. We identified clusters based on overlap between alignment genome coordinates. To select the confident binding sites from the background, we use the “in silico random IP” strategy (*48*). The entire process was repeated 500 times, and false discovery rate (FDR) (*P* value) was determined by counting the observed number of maximum clusters from each of 500 repeats. All observed peaks were assigned an FDR value according to peak height, and peaks with FDR no greater than 0.01 were chosen as a confident binding peak.

### RNA immunoprecipitation–quantitative polymerase chain reaction

TRIzol (Invitrogen, Carlsbad, CA) was used to extract total RNAs from the immunoprecipitate of Csde1 according to the manufacturer’s instructions. Random primers were then used for complementary DNA (cDNA) synthesis. To detect whether Csde1 target genes were significantly and specifically enriched in the Csde1 immunoprecipitate, we used IgG RNA as a reference and performed quantitative reverse transcription polymerase chain reaction (RT-PCR) to determine the relative level of specific RNAs in the IgG and Csde1 immunoprecipitates. Quantitative PCR (qPCR) data represent the mean values from at least three independent experiments. Various genes were selected for PCR amplification in both Csde1 and IgG immunoprecipitates.

### Enrichment analyses for Csde1 RNA binding targets

Gene Ontology and KEGG pathway analyses were performed using the online tool DAVID (http://david.abcc.ncifcrf.gov/). Multiple testing comparisons were performed using the Benjamini-Hochberg FDR method. Enrichment of Csde1-binding targets in ASD-related gene sets was performed using Fisher’s exact test using all protein-coding genes expressed in the mouse brain as the background (*49*).

### Plasmids and antibodies for mouse primary neuron analyses

The full-length *CSDE1* open reading frame (NM_001242891) was purchased from GeneCopoeia (San Diego, CA). *CSDE1* was then cloned into a pCAGGS–IRES–green fluorescent protein (GFP) vector by site-directed mutagenesis (Asc I and Xho I) using the following primers: 5′-TTGGCGCGCCATGGAGAACGTTTTTACT-3′ (forward) and 5′-CCGCTCGAGTTAAGCGTAGTCTGGGACGTCGTATGGGTAGTCAATGACACCAGC-3′ (reverse). The mouse *Csde1* shRNAs were cloned in the pFUGW-H1-GFP vector between the Xba I and Bam HI restriction sites (shRNA1 target: 5′-GCTCTCTGCCCAAAGAAATCA-3′; shRNA2 target: 5′-GCATTACTGAGGAAGCTAATC-3′; NC target: 5′-CCAGATCAGGTGGCAATAAT-3′). The stabilized form of β-catenin construct was from Yu and Malenka (*24*). Lentivirus was provided by Obio Technology (Shanghai, China). The antibodies used in this study included the following: rabbit anti-CSDE1 (1:1000; Abcam, Cambridge, MA; ab113207), rabbit anti-CSDE1 (1:2000; Sigma-Aldrich, St. Louis, MO; HPA018846), mouse anti–SMI 312 (1:500; BioLegend, San Diego, CA; 837904), mouse anti–β-catenin (1:2000; Cell Signaling Technology, Danvers, MA; 2698), rabbit anti-GluR1 (1:1000; Cell Signaling Technology, Danvers, MA; 13185), mouse anti-GAPDH (glyceraldehyde-3-phosphate dehydrogenase) (1:5000; Abcam, Cambridge, MA; ab9484), guinea pig anti-vGlut (1:2000; Millipore, Hessen, Germany; AB5905), rabbit anti-vGAT (1:500; SYSY, Goettingen, Germany; 131002), rabbit anti–α-tubulin (1:5000; Cell Signaling Technology, Danvers, MA; 2148), rabbit anti-GFP (1:500; Invitrogen, Carlsbad, CA; A-11122), and rabbit anti–β-tubulin (1:5000; Sigma, St. Louis, MO; T2200). Appropriate secondary antibodies conjugated with Alexa Fluor dyes anti-mouse or rabbit or guinea pig (1:500 for immunofluorescence and 1:10,000 for Western blot; Jackson ImmunoResearch) were used to detect primary antibodies.

### Mouse primary neuron culture

Embryonic day 15 (E15)–E16 mouse cortical neurons were dissected and plated at a density of 5 × 10^4^ to 20 × 10^4^ cells per well onto coverslips coated with or without poly-d-lysine (0.1 mg/ml; BD Biosciences, San Jose, CA) in plating medium [minimum essential medium (MEM) + 10% fetal bovine serum (FBS); Gibco, Carlsbad, CA]. After 3 to 4 hours, medium was changed to culturing medium (Neurobasal medium + B27 + GlutaMAX; Invitrogen, Carlsbad, CA). Cells were transfected via electroporation with an Amaxa Nucleofector apparatus (Amaxa, Cologne, Germany) at 0 days in vitro (DIV0) according to the manufacturer’s instructions. After DIV5, the cells were fixed for neutric length analyses. For qPCR or Western blot analyses, neurons were infected 2 or 6 days after plating (DIV2 or DIV6) with indicated lentiviruses at a multiplicity of infection of 2, and cells were harvested at DIV6 or DIV15. For dendritic length analyses, neurons were transfected by calcium phosphate transfection at DIV5 and fixed at DIV15 for further analyses. For rescue experiments, we cotransfected each group with three plasmids using a 1:1:0.5 ratio of WT *CSDE1* or *CTNNB1*, shRNAs, and enhanced GFP. For experiments examining the effect of Wnt/β-catenin pathway activation on neurodevelopment in culture, neurons were treated with 5 mM lithium (Sigma, St. Louis, MO) for 7 days. Study protocols complied with all relevant ethical regulations and were approved by the IRB of Central South University.

### Mouse primary neuron RNA extraction and real-time PCR

Total RNA was extracted from cell lysates using TRIzol Reagent (Invitrogen, Carlsbad, CA), and 1 μg was retrotranscribed using the ThermoScript RT-PCR System (Invitrogen, Carlsbad, CA). Real-time PCRs were performed in triplicate on an Applied Biosystems 7900HT Fast Real-Time PCR system using 10 ng of cDNA, 5 μl of SYBR Green PCR Master Mix (Applied Biosystems, Foster City, CA), and 150 nM specific primers (sequences available on request) in a final volume of 10 μl.

### Csde1 immunoblotting

As Csde1 is abundantly expressed in the mouse cortex, cortical tissue homogenates were prepared from mouse brains at different developmental stages. Protein extract of mouse cortical tissue and primary neuronal culture were immersed in radioimmunoprecipitation assay buffer supplemented with a cOmplete protease inhibitor cocktail tablet (Roche, Basel, Switzerland) and sonicated, denatured in 2× SDS loading buffer with 4% β-mercaptoethanol for 10 min at 95°C, and then separated by 12% SDS–polyacrylamide gel electrophoresis. The protein on the gel was transferred to a polyvinylidene fluoride membrane (Immobilon-P; Millipore, Hessen, Germany), and membranes were blocked with 5% nonfat milk in 1× PBST for 1 hour. The membranes were then incubated in primary antibodies overnight at 4°C, washed three times in 1× PBST, and incubated in secondary antibodies for 1 hour at room temperature. The signals were revealed by HRP reaction using SuperSignal Chemiluminescent Substrate (Thermo Fisher Scientific, Waltham, MA).

### Mouse primary neuron immunofluorescence

Cells were washed with 1× PBS three times, fixed in 4% paraformaldehyde (PFA) for 15 min, and blocked in 1× PBS buffer with 5% bovine serum albumin and 0.1% Triton X-100 (PBST) for 30 min at room temperature. The cells were incubated with primary antibodies overnight at 4°C, washed three times in 1× PBS, and then incubated with secondary antibodies at room temperature for 60 min. The signals were observed via fluorescence microscopy.

### Mouse primary neuron phenotype analyses

Images were obtained using a Leica DM5000B fluorescence microscope equipped with a 10× lens (for neurite development in cultured mouse cortical neurons) and a TCS-SP5-II confocal microscope equipped with a 20× objective lens (for dendritic development). To evaluate neurite development in cultured primary cortical neurons, GFP^+^ cells were randomly selected from each condition, and total neurite length was traced with the ImageJ (v1.51j8) software.

For analyses of dendritic development, three-dimensional (3D) reconstructions of entire dendritic processes of each GFP^+^ neuron were obtained from *Z*-series stacks of confocal images. The 2D projection images were traced with ImageJ. The dendrite complexity was analyzed by Sholl analyses. Sholl analysis parameters were as follows: starting radius, 10 μm; ending radius, 120 μm; step size, 10 μm. For the Sholl analysis, concentric circles with an increasing radius (10 μm) were placed around the cell body. The number of intersections of the dendrites and the concentric circles per radial distance from the soma were quantified. Briefly, 8-bit images of cultured neurons were traced using the NeuronJ plugin for ImageJ, and tracing files (*.ndf files) were generated.

Spine density was measured from secondary branches, from the branch point, ranging from 15 to 25 mm in length. The average spine density of specific neurons was analyzed statistically and calculated as the total number of spines per 10 μm of dendritic length of each neuron. The number of synapses was assessed by immunostaining samples with antibodies to excitatory (vGlut) and inhibitory (vGAT) synaptic markers and counting vGlut (or vGAT) and GFP colocalized puncta on dendrites transfected with plasmids. After background subtraction, the threshold value for each channel was automatically adjusted, following an algorithm previously described. The colocalized voxels representing synapses were reported as the number of vGlut (or vGAT) and GFP^+^ puncta per 1 μm. Spine density was measured in Fiji software (ImageJ v1.47b).

Statistics were performed using GraphPad Prism 5.0 software. Compiled data are expressed as means ± SEM. Sample size was not predetermined, but numbers of samples are consistent with previous publications. One- and two-way analysis of variance (ANOVA) followed by either Dunnett’s multiple comparisons test (when comparing to control conditions) or Bonferroni multiple comparisons test was used for experiments with three or more datasets. Equal variances between groups and normal distributions of data were presumed but not formally tested. Molecular and biochemical analyses were performed using a minimum of three biological replicates per condition. *P* < 0.05 was considered statistically significant.

### Whole-cell electrophysiology and data analyses

Voltage-clamp whole-cell recordings were obtained from cultured neurons at room temperature (22° to 25°C). An external solution containing 126 mM NaCl, 2.5 mM KCl, 1.25 mM NaH_2_PO_4_·H_2_O, 26 mM NaCO_3_, 25 mM glucose, 2 mM MgSO_4_, and 2 mM CaCl_2_ (pH adjusted to 7.2 with KOH) was used for the recordings. For recording mEPSCs, glass pipettes with a resistance of 5 to 8 megohms were filled with an internal solution consisting of 120 mM CsMeSO_3_, 15 mM CsCl, 8 mM NaCl, 0.2 mM EGTA, 10 mM Hepes, 2 mM Mg–adenosine triphosphate (ATP), 0.3 mM Na–guanosine triphosphate, 10 mM tetraethylammonium, and 5 mM QX-314 [5-*N*-(2,6-dimethylphenylcarbamoylmethyl) triethylammonium bromide] (290 to 300 mOsm). pH was adjusted to 7.3 with CsOH. Neurons were held at a holding potential of −70 mV. In addition, 1 μM tetrodotoxin and 10 μM bicuculline were added to the external recording solution. For recording mIPSCs, glass pipettes were filled with an internal solution consisting of 140 mM CsCl, 0.1 mM GaCl_2_, 2 mM MgCl_2_, 10 mM Hepes, 0.5 mM EGTA, 4 mM K-ATP, and 5 mM QX-314 (390 to 300 mOsm). pH was adjusted to 7.3 with CsOH. Neurons were held at a holding potential of −70 mV. In addition, 1 μM tetrodotoxin, 10 μM CNQX, and 50 μM D-AP5 were added. The signals were filtered at 2.9 kHz and digitized at 10 kHz using an EPC-10 amplifier and PatchMaster (v2x53) software (HEKA Elektronic Inc., Germany). Recordings with a pipette access resistance of <20 megohms and <20% changes for the duration of recording were included. The mEPSC and mIPSC recordings were analyzed using Clampfit 10.7 software. The frequency and amplitude were measured in each group.

### Fly stocks and generation of transgenic flies

All stocks were cultured in standard medium at 25°C. The w^1118^ strain was used as the WT control in this study. The *Drosophila* Unr hypomorphic mutant line w^1118^; PBac{PB}Unr^c01923^ (FlyBase ID: FBst0010757) was previously mapped and tested (*50*) and was used as a mutant (*dunr*) line in this study. The deficiency strains that removed Unr, Df(3L)BSC815 (abbreviated as Df1) and Df(3L)BSC157 (abbreviated as Df2), were obtained from the Bloomington Stock Center (catalog nos. 27576 and 9544, respectively). UAS-dUnr-RNAi was obtained from Tsinghua Fly Center with the genotype y1sc*v1; P{TRiP.HMS00494}attP2/TM3, Sb1 (catalog no. THU0937). The whole body–expressed Da-Gal4 and pan-neuron–expressed Elav-Gal4 were supplied by Z. Zhang (Center South University, Changsha, Hunan Province, China). Western blotting with rabbit anti-dUnr antibodies was performed to identify the expression level of dUnr in *dunr*, *dunr/Df1*, *dunr/Df2*, and Da-RNAi lines by extracting protein from the whole body of the 4-day-old male flies. Antibody to dUnr was provided from Fatima Gebauer Lab (Centre de Regulacio Genomica, 08003 Barcelona, Spain) (*51*) and used at a dilution of 1:500. Anti–β-actin (A2228, Sigma-Aldrich; 1:5000) was used as loading control. UAS-dUnr and UAS-hCSDE1 transgenic flies were generated by embryo injection into w^1118^ with a recombinant pUAST vector containing the full-length dUnr or hCSDE1 protein-coding sequence, respectively. To generate the dUnr-pUAST vector, the coding sequence for full-length *Drosophila* Unr (dUnr, NM_001300054.1) was obtained by PCR amplification from cDNA and synonymously mutated for resistance to the RNAi fragment of UAS-dUnr-RNAi and then subcloned into the pUAST with a Flag tag on the N terminus. The full-length human *CSDE1* coding sequence (hCSDE1, NM_001242891, GeneCopoeia) was subcloned into the pUAST with the same tag. Genomic DNA PCR and Western blotting with anti-Flag antibody were performed to identify these transgenic lines. Elav-Gal4/+; UAS-dUnr/+; UAS-dUnr-RNAi and Elav-Gal4/+; UAS-hCSDE1/+; UAS-dUnr-RNAi flies were used as dUnr and hCSDE1 rescue lines. Study protocols comply with all relevant ethical regulations and were approved by the IRB of Central South University.

### *Drosophila* NMJ bouton number and fluorescence analyses

Whole-mount immunostaining of the *Drosophila* NMJ was performed essentially as described (*52*). Briefly, wandering third-instar larvae were dissected for body walls in HL3 buffer [70 mM NaCl, 5 mM KCl, 20 mM MgCl_2_·6H_2_O, 10 mM NaHCO_3_, 115 mM sucrose, 5 mM trehalose, 5 mM Hepes (pH 7.3)]. After fixing with ice methanol for 10 min and washing with 0.2% PBST four times for 10 min each, the samples were incubated with primary antibody at 4°C overnight, followed by appropriate secondary antibodies for 3 hours at room temperature. The antibodies were diluted in blocking agent (0.2% PBST with 5% normal goat serum), and these primary and secondary antibodies were used: rabbit anti-HRP (1:1000; code number: 323-005-021; Jackson ImmunoResearch, West Grove, PA), mouse anti-DLG [4F3; 1:500; Developmental Studies Hybridoma Bank (DSHB)], mouse anti-BRP (nc82; 1:25; DSHB), mouse anti-GluRIIA (8B4D2; 1:25; DSHB), and Alexa Fluor 488– or cy3-conjugated anti-mouse and anti-rabbit secondary antibodies (1:500; Jackson ImmunoResearch, West Grove, PA). Following the antibody incubation, the samples were washed extensively and mounted on slides with VectaShield antifade mounting medium (Vector Laboratories, catalog: H-1000) and sealed with fingernail polish. Type Ib boutons at muscle 4 of abdominal segment 3/4 were imaged. Pictures were collected using a Leica TCS SP5 confocal microscope. For bouton number analysis, type Ib bouton numbers and satellite bouton numbers were defined as previously described (*53*). For fluorescent intensity comparisons, the sum of pixel intensity in each channel was recorded by ImageJ software (National Institutes of Health), and the anti-HRP staining was used as control. The ratio of anti-BRP/anti-HRP and anti-GluRIIA/anti-HRP staining intensity was calculated for each genotype, and different genotypes were normalized to the control in each experiment set. Statistical analyses were performed using GraphPad Prism 5.0 software (GraphPad Software Inc.). Statistical analysis was performed using two-tailed Student’s *t* tests for comparisons of two group means. Data are presented as means ± SEM. A *P* value of <0.05 was considered to be statistically significant. The analyses were double blinded.

### *Drosophila* FM1-43 dye labeling

Procedures for the fluorescent dye FM 1-43 assay were described previously (*54*). Wandering third-instar larvae were dissected for body walls in modified HL-3 Ca^2+^-free solution: 70 mM NaCl, 5 mM KCl, 20 mM MgCl_2_, 10 mM NaHCO_3_, 5 mM trehalose, 115 mM sucrose, and 10 mM Hepes (pH 7.2). For high K^+^ stimulation, the samples were incubated with 4 μM FM1-43 dye (Invitrogen, F35355) for 5 min in modified HL-3 solution containing 90 mM KCl and 1.5 mM CaCl_2_ [110 mM NaCl, 90 mM KCl, 10 mM MgCl_2_, 1.5 mM CaCl_2_, 30 mM sucrose, 5 mM Hepes, 5 mM trehalose, and 10 mM NaHCO_3_ (pH 7.2)] and then washed with modified HL-3 Ca^2+^-free solution. The samples were then fixed with 4% PFA for 35 min and washed three times with PBS before mounting for images. Zeiss LSM5 confocal microscope was used to take images with a 40× objective. Fluorescence intensities were calculated with ImageJ software (National Institutes of Health) and normalized to the average loading fluorescence intensity in controls within the same experimental set.

### *Drosophila* NMJ electrophysiology analyses

Conventional intracellular recordings for assessing NMJ synaptic transmissions were carried out as previously described (*55*). Briefly, wandering third-instar larvae were dissected in calcium-free HL3.1 saline and recorded in HL3.1 saline containing 0.6 mM Ca^2+^ using intracellular microelectrodes (10 to 20 megohms) filled with 3 M KCl. Recordings were performed at 20° to 22°C with an Axoclamp 2B amplifier (Molecular Devices) in bridge mode, and the recorded data were processed with pCLAMP version 10.2 software (Molecular Devices). Both EJPs and mEJPs were recorded from muscle 6 of abdominal segment A3. EJPs were evoked by a Grass S48 stimulator (Astro-Grass Inc.) with suprathreshold stimulating pulses at 0.3 Hz, three EJP responses were collected for each animal, and mEJPs were recorded for a period of 60 s after the EJP recording. The data for analyses were recorded from cells with resting membrane potentials ranging from −60 to −65 mV.

## Supplementary Material

http://advances.sciencemag.org/cgi/content/full/5/9/eaax2166/DC1

Download PDF

Table S1

Disruptive variants of CSDE1 asociate with autism and interfere with neuronal development and synaptic transmision

## SUPPLEMENTARY MATERIALS

Supplementary material for this article is available at http://advances.sciencemag.org/cgi/content/full/5/9/eaax2166/DC1 Fig. S1. Mean coverage of the coding regions of *CSDE1* in ExAC whole-exome sequencing data. Fig. S2. Correlations of the two independent HITS-CLIP experiments and pathway enrichment of Csde1-binding targets. Fig. S3. Time-spatial expression pattern of CSDE1 in human and mouse brain. Fig. S4. CSDE1 disruptive mutations show loss of function. Fig. S5. Interfere efficiency of two shRNA in neurons. Fig. S6. Immunoblotting with anti-dUnr antibodies was performed to examine the expression level of dUnr in *dunr*, *dunr/Df1*, *dunr/Df2*, and Da-RNAi lines. Fig. S7. Overexpression of dUnr or hCSDE1 has no effect on both bouton number and satellite bouton number compared to WT controls. Table S1. Detailed clinical information for probands or carrier patients with LGD mutation or de novo missense mutations. Table S2. Validation result of selected RNA binding targets. Table S3. High-confidence RNA binding targets called by two software programs in two experiments.
