# Supplementary material for: Disruptive variants of CSDE1 associate with autism and interfere with neuronal development and synaptic transmission
Source: Sci Adv. 2019 Sep 25;5(9):eaax2166. doi: 10.1126/sciadv.aax2166 (PMC6760934; doi:10.1126/sciadv.aax2166)
Supplement: http://advances.sciencemag.org/cgi/content/full/5/9/eaax2166/DC1 [file supp_5_9_eaax2166__index.html]

Science Advances | Science AdvancesAAASSearchScience AdvancesMenu

## Supplementary Materials

**The PDF file includes:**

- Fig. S1. Mean coverage of the coding regions of *CSDE1* in ExAC whole-exome sequencing data.
- Fig. S2. Correlations of the two independent HITS-CLIP experiments and pathway enrichment of Csde1-binding targets.
- Fig. S3. Time-spatial expression pattern of CSDE1 in human and mouse brain.
- Fig. S4. CSDE1 disruptive mutations show loss of function.
- Fig. S5. Interfere efficiency of two shRNA in neurons.
- Fig. S6. Immunoblotting with anti-dUnr antibodies was performed to examine the expression level of dUnr in *dunr*, *dunr/Df1*, *dunr/Df2*, and Da-RNAi lines.
- Fig. S7. Overexpression of dUnr or hCSDE1 has no effect on both bouton number and satellite bouton number compared to WT controls.

Download PDF

**Other Supplementary Material for this manuscript includes the following:**

- Table S1 (Microsoft Excel format). Detailed clinical information for probands or carrier patients with LGD mutation or de novo missense mutations.
- Table S2 (Microsoft Excel format). Validation result of selected RNA binding targets.
- Table S3 (Microsoft Excel format). High-confidence RNA binding targets called by two software programs in two experiments.

**Files in this Data Supplement:**

- Adobe PDF - aax2166\_SM.pdf
