# Supplementary material for: Disruptive variants of CSDE1 associate with autism and interfere with neuronal development and synaptic transmission
Source: Sci Adv. 2019 Sep 25;5(9):eaax2166. doi: 10.1126/sciadv.aax2166 (PMC6760934; doi:10.1126/sciadv.aax2166)
Supplement: Download PDF [file aax2166_SM.pdf]

Science Advances
AAAS

advances.sciencemag.org/cgi/content/full/5/9/eaax2166/DC1

Supplementary Materials for

Disruptive variants of *CSDE1* associate with autism and interfere with neuronal development and synaptic transmission

Hui Guo*, Ying Li, Lu Shen, Tianyun Wang, Xiangbin Jia, Lijuan Liu, Tao Xu, Mengzhu Ou, Kendra Hoekzema, Huidan Wu, Madelyn A. Gillentine, Cenying Liu, Hailun Ni, Pengwei Peng, Rongjuan Zhao, Yu Zhang, Chanika Phornphutkul, Alexander P. A. Stegmann, Carlos E. Prada, Robert J. Hopkin, Joseph T. Shieh, Kirsty McWalter, Kristin G. Monaghan, Peter M. van Hasselt, Koen van Gassen, Ting Bai, Min Long, Lin Han, Yingting Quan, Meilin Chen, Yaowen Zhang, Kuokuo Li, Qiumeng Zhang, Jieqiong Tan, Tengfei Zhu, Yaning Liu, Nan Pang, Jing Peng, Daryl A. Scott, Seema R. Lalani, Mahshid Azamian, Grazia M. S. Mancini, Darius J. Adams, Malin Kvarnung, Anna Lindstrand, Ann Nordgren, Jonathan Pevsner, Ikeoluwa A. Osei-Owusu, Corrado Romano, Giuseppe Calabrese, Ornella Galesi, Jozef Gecz, Eric Haan, Judith Ranells, Melissa Racobaldo, Magnus Nordenskjold, Suneeta Madan-Khetarpal, Jessica Sebastian, Susie Ball, Xiaobing Zou, Jingping Zhao, Zhengmao Hu, Fan Xia, Pengfei Liu, Jill A. Rosenfeld, Bert B. A. de Vries, Raphael A. Bernier, Zhi-Qing David Xu, Honghui Li, Wei Xie, Robert B. Hufnagel*, Evan E. Eichler*, Kun Xia*

*Corresponding author. Email: guohui@sklmg.edu.cn (H.G.); robert.hufnagel@nih.gov (R.B.H.); eee@gs.washington.edu (E.E.E.); xiakun@sklmg.edu.cn (K.X.)

Published 25 September 2019, *Sci. Adv.* **5**, eaax2166 (2019)
DOI: 10.1126/sciadv.aax2166

The PDF file includes:

Fig. S1. Mean coverage of the coding regions of *CSDE1* in ExAC whole-exome sequencing data.
Fig. S2. Correlations of the two independent HITS-CLIP experiments and pathway enrichment of Csde1-binding targets.
Fig. S3. Time-spatial expression pattern of CSDE1 in human and mouse brain.
Fig. S4. CSDE1 disruptive mutations show loss of function.
Fig. S5. Interfere efficiency of two shRNA in neurons.
Fig. S6. Immunoblotting with anti-dUnr antibodies was performed to examine the expression level of dUnr in *dunr*, *dunr/Df1*, *dunr/Df2*, and Da-RNAi lines.
Fig. S7. Overexpression of dUnr or hCSDE1 has no effect on both bouton number and satellite bouton number compared to WT controls.

Other Supplementary Material for this manuscript includes the following:

(available at advances.sciencemag.org/cgi/content/full/5/9/eaax2166/DC1)

Table S1 (Microsoft Excel format). Detailed clinical information for probands or carrier patients with LGD mutation or de novo missense mutations.
Table S2 (Microsoft Excel format). Validation result of selected RNA binding targets.

Table S3 (Microsoft Excel format). High-confidence RNA binding targets called by two software programs in two experiments.

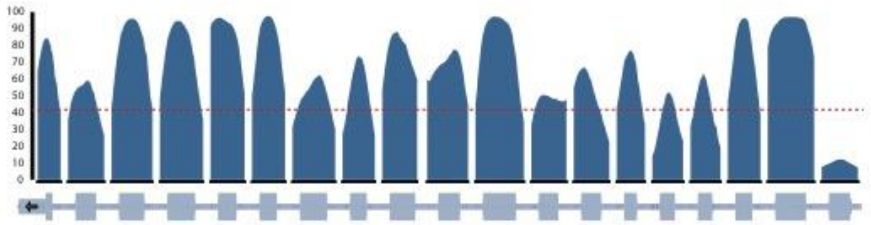

Fig. S1. Mean coverage of the coding regions of *CSDE1* in ExAC whole-exome sequencing data. The overall mean coverage is 64.39. Majority of the coding nucleotide sites has a mean coverage over 40× (red line).

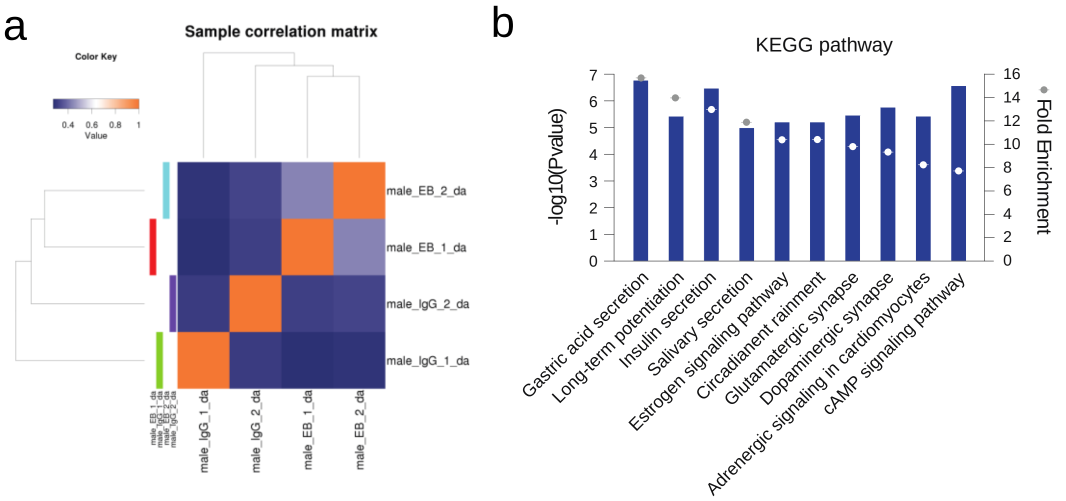

Fig. S2. Correlations of the two independent HITS-CLIP experiments and pathway enrichment of Csde1-binding targets. a, The correlations of the two independent HITS-CLIP experiments (male_EB_1_da, male_EB_2_da,) and their corresponded controls (male_IgG_1_da, male_IgG_2_da). **b,** Bar plot shows Csde1-binding targets enriched in synapse plasticity related pathways and several secretory pathways. Top ten significant pathways were showed.

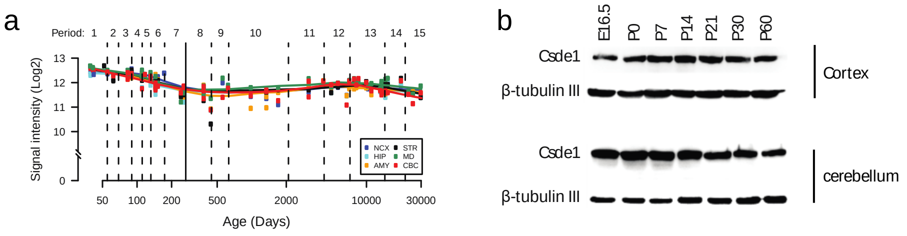

Fig. S3. Time-spatial expression pattern of CSDE1 in human and mouse brain. Time-spatial expression pattern of *CSDE1* mRNA (left) in human brain and Csde1 protein (right) in mouse brain. The mRNA time-spatial expression pattern was generated from Human Brain Transcriptome database (http://hbatlas.org/).

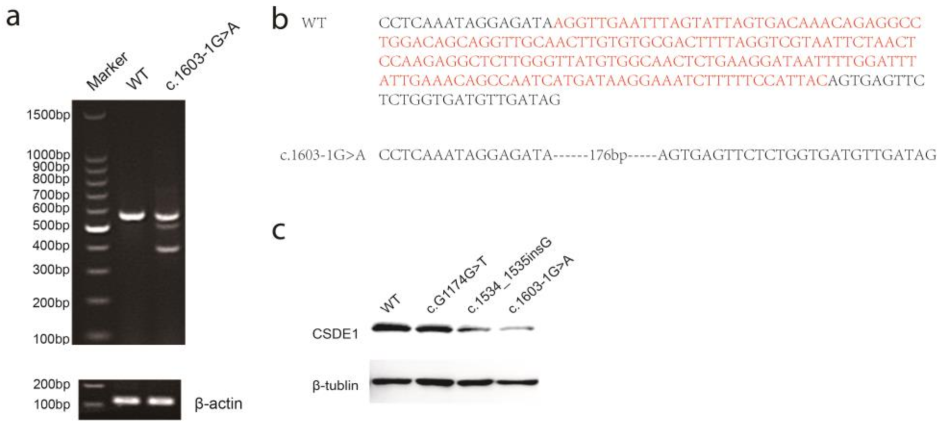

Fig. S4. CSDE1 disruptive mutations show loss of function. a, Agarose gel electrophoresis shows three cDNA bands in the patient with splicing mutation c.1603-1G>A. **b**, Sanger sequencing confirmed a 176bp deletion by the c.1603-1G>A mutation. **c**, Immunoblotting of CSDE1 in lymphocyte cells derived from the three patients from ACGC cohort revealed that CSDE1 expression were dramatically decreased.

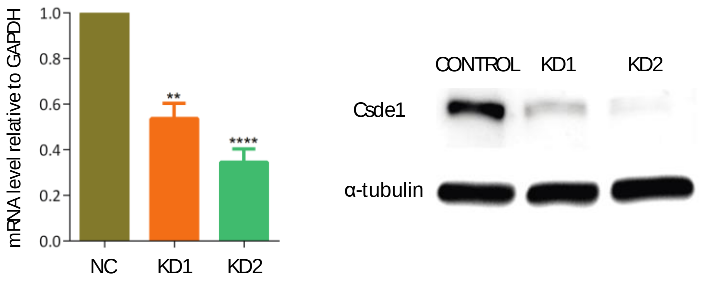

Fig. S5. Interfere efficiency of two shRNA in neurons. The bar plot on the left shows the statistic result.

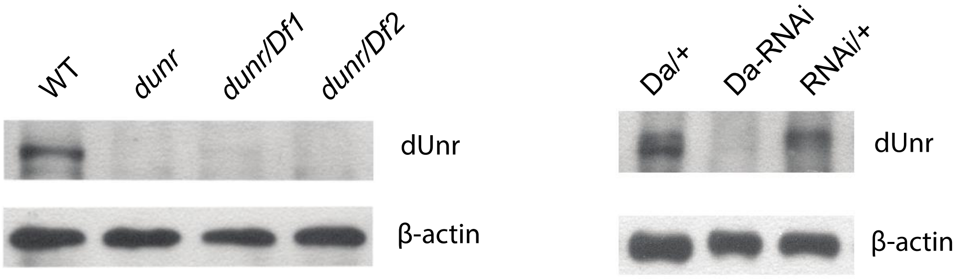

Fig. S6. Immunoblotting with anti-dUnr antibodies was performed to examine the expression level of dUnr in *dunr, dunr/Df1, dunr/Df2,* and Da-RNAi lines. WT, Da/+ and RNAi/+ were controls. β-actin was used as loading control.

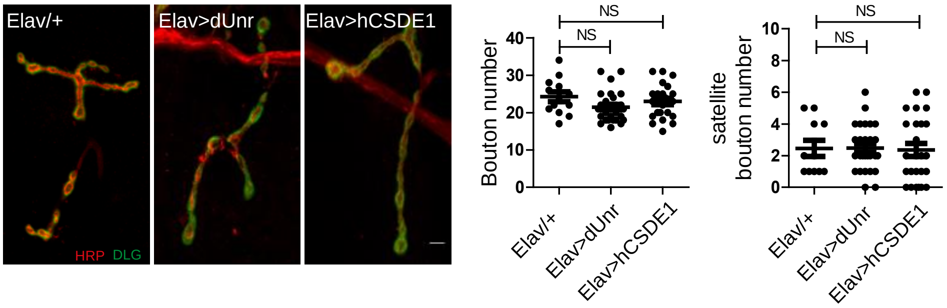

Fig. S7. Overexpression of dUnr or hCSDE1 has no effect on both bouton number and satellite bouton number compared to WT controls.
